# Editorial: Advancements in synthetic microbiomes for enhancing animal health

**DOI:** 10.3389/fvets.2025.1708299

**Published:** 2025-10-23

**Authors:** Zhengge Zhao, Xiaoyuan Wei, Feilong Deng, Shuli Yang, Ying Li, Jianmin Chai

**Affiliations:** ^1^Guangdong Provincial Key Laboratory of Animal Molecular Design and Precise Breeding, School of Animal Science and Technology, Foshan University, Foshan, China; ^2^Department of Internal Medicine, Morsani College of Medicine, University of South Florida, Tampa, FL, United States

**Keywords:** microbiome consortium, assembly of microbes, microbiota, host–microbe interactions, microbial modulation

The study of microbial communities is grounded in two foundational, yet distinct, concepts. The microbiota refers to the assembly of microorganisms belonging to different kingdoms, while the microbiome not only refers to the microorganisms involved but also encompass their theater of activity, forming a dynamic ecosystem integrated into macro-ecosystems like eukaryotic hosts (1). Microbiomes exhibit an astounding genetic, physiological, and biochemical diversity, enabling their survival across diverse environmental conditions and in various hosts worldwide. With advances in sequencing technologies, our understanding of microbiomes' abilities and their effects on hosts and environments has significantly expanded (2). For instance, recent research on the giant panda gut microbiome has revealed that *Streptococcus lactis* serves as a key player in the gut microbiota, enhancing protein metabolism to help the host adapt to a high-fiber, low-protein bamboo diet (3). Additionally, the identification of 1,214 antibiotic resistance genes (ARGs) in the giant panda gut microbiome that are homologous to those found in the human gut highlights a potential risk of cross-species transmission between giant pandas and humans (4). Researchers are now harnessing the expansive capabilities of microbes for bioremediation and various applications in biotechnology, agriculture, and medicine. By constructing synthetic microbial communities—novel in composition, genetics, and phenotypes—scientists can tackle foundational biological issues and address broader societal challenges. Nonetheless, expansive research is essential to thoroughly explore the significance and applications of synthetic microbiomes.

This Research Topic aims to collect state-of-the-art knowledge regarding the manipulation of synthetic microbiomes, particularly focusing on animal gut microbiota. The gut microbiome is pivotal in nutrient metabolism, immune response, and the overall wellbeing of animals (5, 6). Defined as engineered collections of microbes with unique or intentionally modified characteristics, synthetic microbiomes allow researchers to investigate how these communities can enhance nutrient absorption, prevent metabolic diseases, and boost animal health by influencing digestion and metabolism processes. Key to this research is also the understanding of interactions between synthetic and natural microbiota components.

The research included in this special topic reveals the application value of synthetic microbiomes in animal health from multiple perspectives. The first category of research focuses on microbial assembly and functional regulation associating with animal health. For example, Zhang et al. employed a combination of *in vitro* and *in vivo* approaches to evaluate the effects of eight plant-derived fermentation broths (FB) on rumen fermentation, gastrointestinal development, and microbial communities in fattening lambs. Their results demonstrated that adding FB to drinking water at a 1:500 ratio improved rumen fermentation and microecological balance. Wang W. et al. revealed that ruminal methane production is determined by functional pathway activity rather than archaeal abundance, with Treponema species showing a consistent negative correlation with methane emission—highlighting their potential as probiotics for methane mitigation. Fan et al. showed that yeast peptides significantly reduced the incidence of diarrhea in lambs by targeted modulation of the colonic microbiota, with a recommended dosage of 2,000 mg/d. In a study on 200-day-old Hyline Brown laying hens, Wang Y. et al. found that dietary supplementation with compound probiotics enhanced production performance and immune function by optimizing gut microbial composition. Using a male C57BL/6 mouse model, Moon et al. reported that both 4% bitter melon and 4% fermented bitter melon significantly altered the gut microbiota and regulated metabolism, suggesting their potential use in preventing diet-induced metabolic disorders and obesity. Yang et al. evaluated the effects of replacing chemical fertilizers with different concentrations of biogas slurry (BS) on the microbial community structure and gene distribution in rapeseed field soil. Their short-term study indicated minimal impact on soil microbes and antibiotic resistance genes, supporting BS as a feasible alternative to chemical fertilizers, though long-term ecological safety under varying soil types and management practices requires further monitoring. Lee et al. observed that antioxidant supplementation induced significant molecular changes in the liver—particularly in transcriptional activity and mitochondrial processes—even in the absence of detectable phenotypic differences in Hanwoo beef cattle. Additionally, Wang Y. et al. systematically reviewed the role of the gut microbiota in pig health and productivity, noting that there is still no unified definition of an “optimal” or “healthy” microbial community. They emphasized that future research should focus on microbial modulators and their physiological and immune functions to develop strategies for rapidly restoring microbial balance after stress, antibiotic treatment, or infection, thereby improving productivity, reducing losses, and preventing diseases.

The second category explores microbiome engineering under stress conditions. Under heat stress, Mei et al. found that niacin supplementation improved poultry performance by enriching beneficial microorganisms and promoting short-chain fatty acid production. Mohiti-Asli et al. reported that lysophospholipid supplementation enhanced production performance and lipid utilization in heat-stressed broilers. Wang X. et al. demonstrated that a Chinese herbal formula improved physiological and biochemical parameters, enhanced antioxidant and immune capacity, and alleviated heat stress through modulation of the rumen microbiota.

In addition, several studies address mechanistic insights and innovative applications of microbiomes. Wang Y. et al. investigated crAss-like phages in the pig intestine, revealing intricate virus–bacterium interaction mechanisms. Cantas et al. provided evidence that fecal microbiota transplantation (FMT) is a simple and safe procedure that can restore gut microecological balance and improve overall health in dogs. Zhao et al. elucidated the host–bacterium interactions during single and co-infection of mammary epithelial cells with *Escherichia coli* or *Staphylococcus aureus*, uncovering distinct and shared transcriptional regulatory networks that provide a theoretical basis for precise prevention and control of mastitis.

These studies demonstrate that we can effectively address challenges related to animal health, production, and the environment through precise modulation of microbiomes. However, translating laboratory findings into practical applications still faces challenges such as stability and safety. Research on synthetic microbiomes is in a phase of rapid development, holding significant potential for promoting animal health and achieving sustainable development in animal husbandry. The findings presented in this Research Topic provide essential knowledge and innovative ideas for the future advancement of this field.
